# The Research on the Handwriting Stability in Different Devices and Conditions

**DOI:** 10.3390/s26020538

**Published:** 2026-01-13

**Authors:** Hsiang-Ju Lai, Long-Huang Tsai, Kung-Yang Hsu, Wen-Chao Yang

**Affiliations:** 1 Forensic Science Division, Ministry of Justice Investigation Bureau, New Taipei City 231209, Taiwan; raylin@mjib.gov.tw; 2 Forensic Science Section, Keelung City Police Bureau, Keelung City 201201, Taiwan; fs821213@klg.gov.tw; 3 Department of Forensic Science, Central Police University, Taoyuan City 333322, Taiwan; una466@mail.cpu.edu.tw

**Keywords:** Digital Capture Signature, handwriting, Dynamic Time Warping, Android, iOS

## Abstract

**Highlights:**

The purpose of this study is to conduct a rigorous and systematic investigation into the emerging field of digital handwriting identification and to establish a representative, large-scale digital handwriting dataset. In parallel, it aims to develop advanced technologies and tools to enhance sample quality, thereby providing a robust technical foundation for future document examination and promoting the continued evolution and application of forensic science in the digital era.

**What are the main findings?**

- A new handwriting dataset, comprising 16,500 handwriting DCSs, including Chinese characters, English characters, and numbers, has been constructed.
- The use of styluses provides more precise distinctions between same- and different-writer samples compared with direct finger-based writing.

**What are the implications of the main findings?**

- In most cases, FastDTW demonstrated better separation the SC-DTW method used for on-line signature verification under the examined conditions.
- As with the ENFSI Best Practice Manual for Forensic Handwriting Examination, it is highly recommended to obtain reference handwriting samples in documentation examination using the same writing equipment or under the same writing conditions.

**Abstract:**

With the rapid advancement of technology in recent years, signatures on contracts and documents have increasingly shifted from traditional handwritten forms on paper to digital handwritten signatures executed on devices (hereafter referred to as digital tablets). This transition introduces new challenges for forensic document examination due to the differences in writing instruments. According to the European Network of Forensic Science Institutes (ENFSI), a Digital Capture Signature (DCS) refers to data points captured during the writing process on digital devices such as tablets, smartphones, or signature pads. In addition to retaining the visual image of the signature, DCS provides more information previously unavailable, including pen pressure, stroke order, and writing speed. These features possess potential forensic value and warrant further study and evaluation. This study employs three devices—Samsung Galaxy Tab S10, Apple iPad Pro, and Apple iPad Mini—together with their respective styluses as experimental tools. Using custom-developed handwriting capture software for both Android and iOS platforms, we simulated signature-writing scenarios common in the financial and insurance industries. Thirty participants were asked to provide samples of horizontal Chinese, English, and number writings (FUJ-IRB NO: C113187), which were subsequently normalized and segmented into characters. For analysis, we adopted distance-based time-series alignment algorithms (FastDTW and SC-DTW) to match writing data across different instances (intra- and inter-writer). The accumulated distances between corresponding data points, such as coordinates and pressure, were used to assess handwriting stability and to study the differences between same-writer and different-writer samples. The findings indicate that preprocessing through character centroid alignment, followed by the analysis, substantially reduces the average accumulated distance of handwriting. This procedure quantifies the stability of an individual’s handwriting and enables differentiation between same-writer and different-writer scenarios based on the distribution of DCS distances. Furthermore, the use of styluses provides more precise distinctions between same- and different-writer samples compared with direct finger-based writing. In the context of rapid advancements in artificial intelligence and emerging technologies, this preliminary study aims to contribute foundational insights into the forensic application of digital signature examination.

## 1. Introduction

Signatures have long been a cornerstone of identity verification in legal and financial transactions. As technology advances, there is a notable shift from traditional pen-and-paper signatures to digital handwritten signatures, which are executed on electronic devices such as tablets and signature pads. This transition introduces new opportunities and challenges for forensic document examiners. Unlike static ink signatures, a digitally captured signature (DCS) [1] not only preserves the visual trace of the signature but also records dynamic data from the signing process. During a DCS event, the signing device logs a time series of pen movements, including the X–Y coordinates of the stylus tip, pressure applied, and timing information, collectively referred to as channels in ISO/IEC 19794-7 [2]. According to ISO/IEC 19794-7 [2], such signature time-series data can be stored in a standardized format comprising multiple channels (e.g., pen position, pressure, timestamp). These rich data offer additional features (such as stroke order, speed, and rhythm) that were previously unavailable in off-line scanned signatures. From a forensic standpoint, these dynamic features hold potential evidentiary value, as they capture aspects of the signatory’s neuromuscular coordination and behavior that are difficult for an impostor to mimic [1].

International forensic science communities have begun to recognize and formalize the treatment of digitally captured handwriting. The European Network of Forensic Science Institutes (ENFSI) Best Practice Manual for Forensic Handwriting Examination [1] explicitly defines a digitally captured signature (DCS) as a handwritten signature digitized through the chronological sampling of writing movements, consisting of a series of data points [1]. Terms such as biometric signature, dynamic signature, on-line signature, or electronic handwritten signature also refer to these types of signatures. In essence, a DCS is an on-line signature, captured in real time, as opposed to an off-line (scanned) signature image. These definitions underscore that, while the format of signatures may evolve, the fundamental goal in forensic examination remains the same: to determine whether two signatures originated from the same writer.

Traditional standards for forensic handwriting examination, developed initially for ink signatures, are being adapted to encompass these new digital formats. The ASTM Standard Guide for Examination of Handwritten Items (ASTM E2290-07) provided foundational procedures for comparing questioned and known handwriting specimens [3]. More recently, the ANSI/ASB Standard 070 (1st ed., 2022) updates these guidelines and acknowledges the advent of electronically captured signatures. It notes that the examination of the pictorial image of a DCS (the visual trace) can generally follow the same procedures as for pen-and-paper signatures [4]. However, it cautions that the on-screen rendering of a DCS may exhibit lower quality or distortion compared to ink on paper, and importantly, that the underlying data (the recorded coordinates, timing, and pressure) could prove helpful in analysis—although analysis of such raw data is beyond the scope of the standard’s current edition [4]. This reflects a recognition that new methodologies are needed to fully leverage DCS data in forensic examinations. Likewise, the ENFSI manual includes dedicated appendices for handling DCS evidence, covering topics such as proper data handling, understanding the capture process, and analytical approaches for DCS comparison. These guidelines encourage forensic handwriting experts to collaborate with digital evidence specialists and remain aware of how hardware/software differences may affect the signature data.

Despite these emerging guidelines, practical research on the stability of DCS and the reliability of identifying individuals from such data remains in its early stages of development. A key question for examiners is whether a person’s digital signature captured on different devices remains consistently identifiable. Heckeroth et al. compared the features of DCS to those of traditional signatures to determine their similarities and differences [5]. Magnier et al. proposed a new supervised edge map quality measure for the overall evaluation of contour map quality, taking into account the number of false positives and false negatives [6]. Zimmer et al. examined the challenges of comparing signatures collected on different hardware/software setups [7]. For example, one device might sample coordinates at 100 Hz and record pressure on a 0–1023 scale, whereas another device uses a sampling rate of 200 Hz and a pressure scale of 0–4095. Furthermore, the writing surface and stylus of each device can affect how a person writes (e.g., friction, latency). These factors mean that an examiner comparing a signature captured on an iPad with one on a commercial signature pad must be cautious—some differences might stem from the device rather than the writer. Standardization efforts, such as ISO/IEC 19794-7, help by providing a standard format to store data, but they do not, by themselves, resolve hardware-related discrepancies in the dynamic profile. Best practice guidelines [1] recommend that forensic handwriting examiners be aware of the data capture conditions and, if possible, obtain reference signatures under similar conditions to the questioned signature. They also underscore the importance of preserving the raw signature data (the time series) for analysis, not just the static image, as dynamic data can offer supporting evidence in a forensic comparison. actors such as screen size, sampling rate, and stylus characteristics might influence the feature quality of the captured signatures.

Another active area of research is the development of robust computational methods for comparing and verifying signatures. In the domain of biometric authentication, on-line signature verification has been studied for decades, yielding a variety of algorithms for measuring the similarity between two signature time series. Classic approaches include probabilistic models, such as the Longest Common Subsequence (LCS) matching [8] and Hidden Markov Models (HMMs) [9,10,11], as well as distance-based template matching techniques [12,13]. Lai et al. [14] introduced a novel feature descriptor, the length-normalized path signature (LNPS), which is used for feature representation before being input into a Gated Recurrent Unit (GRU) network. The network is trained using triplet loss and center loss optimized via backpropagation. Tolosana et al. [15] proposed an approach that further extracts 23 hand-crafted temporal features and employs bidirectional Long Short-Term Memory (BiLSTM) and GRU networks within a Siamese architecture to learn a dissimilarity metric between pairs of signatures. Among the latter, Dynamic Time Warping (DTW) has emerged as a simple yet powerful algorithm for sequence alignment and similarity measurement [13,16,17,18,19]. DTW dynamically aligns two time series by allowing non-linear stretching of the time axis, finding an optimal path that minimizes the cumulative distance between the elements of the sequences. It has been successfully applied to signature data as early as the mid-2000s—for example, Lei and Govindaraju used DTW to examine the consistency of signature features and reported that certain dynamic features remain relatively stable for the same individual across samples [19]. However, the classic DTW algorithm has quadratic time complexity; many ideas have been introduced to reduce its amortized time or to approximate it quickly. One of the most cited approximate approaches is FastDTW [20]. Subsequent studies have refined DTW, introducing variations such as weighted DTW or region-based DTW to enhance discrimination between genuine and forged signatures. A notable extension is the concept of stability regions within a signature. Parziale et al. proposed Stability-Modulated DTW (SM-DTW), which weights the DTW alignment cost to emphasize inherently stable segments of the signature, yielding better verification accuracy [21].

In recent years, there has also been interest in combining DTW with shape-based descriptors. Shape Context Dynamic Time Warping (SC-DTW) augments the time-warping algorithm by incorporating shape context features at each time point [22]. Instead of comparing raw point coordinates or pen velocities directly, SC-DTW computes a shape context descriptor, which is essentially a histogram that captures the spatial distribution of points around a given pen-tip position, and uses these descriptors to guide the alignment. Jia et al. employed SC-DTW as part of a two-stage on-line signature verification system, achieving an Equal Error Rate of 2.39% on a public signature dataset, which is competitive with state-of-the-art machine learning methods [23]. These advances highlight that, even as deep learning techniques (e.g., CNNs or RNNs, as used in works like Tolosana et al. [24]) are emerging for signature verification, distance-based algorithms like DTW and SC-DTW remain highly relevant. They offer interpretability and efficiency, which are valuable in forensic contexts where examiners must often explain their reasoning and work with limited enrollment samples.

In this study, we aim to bridge the gap between these technical developments and forensic applications by conducting a comprehensive evaluation of DCS data across different devices and writing conditions. Specifically, we focus on measuring: (1) how consistently a given individual can reproduce their signature on digital devices (intra-writer stability), (2) how well we can distinguish different individuals based on their DCS (inter-writer variability), and (3) whether the choice of input device (including hardware and stylus differences) affects the capture quality and comparison results. We employ FastDTW and SC-DTW to quantify signature differences, given their proven track record in signature analysis. The study is conducted with a view toward forensic implications—the ultimate goal is to inform examiners and standard setters whether such quantitative measures can augment traditional visual examination, and how factors such as device variation should be accounted for. By aligning our investigation with international standards (ISO/IEC 19794-7 [2] data format) and forensic best practices (ANSI/ASB 070-2022 [4], ASTM E2290-07 [3], ENFSI BPM-FHX-01 [1]), we also discuss how the outcomes can be integrated into current forensic protocols and where further developments or guidelines may be needed.

## 2. Materials and Methods

### 2.1. Data Collection

The study involved thirty volunteer participants who provided multiple handwriting samples under various conditions (FUJ-IRB NO: C113187). Each participant was asked to produce signatures and handwriting samples in three different content categories: Chinese, English, and digits. For the Chinese writing, a short phrase (two to four characters in length, written in traditional Chinese script) was used; for English, participants signed their name or a simple word; for digits, participants wrote a sequence of numerals (e.g., “0 1 2 3 4 5 6 7 8 9”). Each participant repeated each category 10 times, resulting in 30 samples per participant (10 per category). To study the effect of the input device, writing was performed on three different digital devices: an Apple iPad Pro manufactured in China, an Apple iPad Mini manufactured in China, and a Samsung Galaxy Tab S10 Ultra tablet manufactured in Korea. Each device was paired with its respective active stylus (Apple Pencil for the iPad mini, Apple Pencil Pro for the iPad Pro, and the S-Pen stylus for the Samsung tablet). Participants were instructed to mimic a natural signing scenario for each sample, as if signing a document or form on that device.

**Figure 1:**
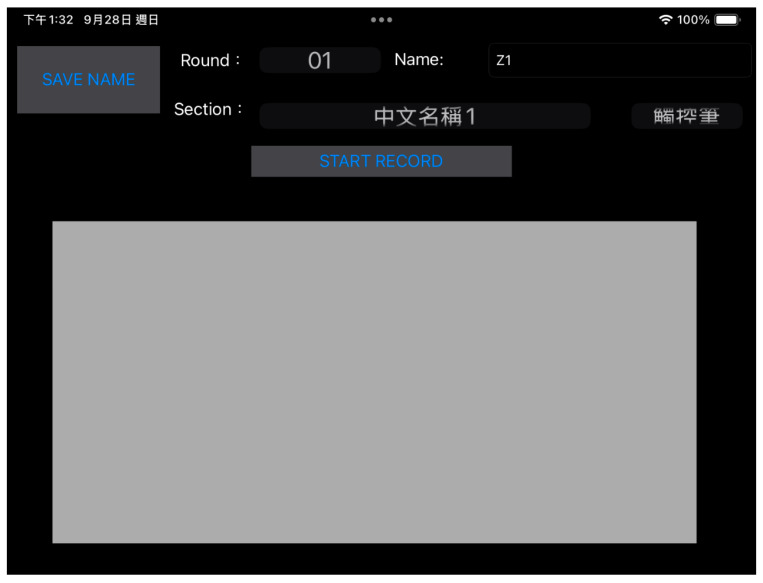
The writing area on the Apple iPad mini.

Two custom handwriting capture applications were developed for each platform (iOS and Android) to record the signatures. The apps provided a uniform writing canvas (approximately postcard-sized on each tablet screen) with coordinate systems calibrated to each screen’s resolution. For example, on the iPad mini, the writing area corresponded to a physical size of approximately 100 × 50 mm on screen, with a pixel resolution of about 1280 × 640 for data capture. The section (writing type) is “Chinese Name 1” with a “stylus”, as shown in Figure 1. The sampling rate for the stylus signals was approximately 100 Hz on the iPad devices (the iPad Pro with ProMotion could sample at up to 120 Hz, while the iPad Mini was limited to 60–120 Hz, depending on the model), and similarly around 60–100 Hz on the Samsung tablet. Each recorded sample (signature) was saved as a time-series data file containing sequential records of the pen position and other parameters. Table 1 (below) outlines the typical data format and units, consistent with ISO/IEC 19794-7:2021 [2]: for each sampled point in time, the X coordinate, Y coordinate, the altitude angle, and the azimuth angle of the pen tip on the screen (in device-specific units or normalized units) were stored, along with the pressure level (a dimensionless unit from the device’s API, typically ranging 0 to a maximum value such as 1.0 or 4095), and a timestamp or sample index.

### 2.2. Preprocessing

To mitigate the impact of the handwriting scale and shift, and to extract information from DCS, the preprocessing process is necessary. The preprocessing process includes three steps: segmentation, feature extraction, and z-score standardization.

#### 2.2.1. Segmentation

Each sample, whether in Chinese or English, that contained multiple characters was automatically segmented into individual characters upon collection. Segmentation was achieved by analyzing pen lift events and spacing. When the pen was lifted and moved a significant distance, it was assumed to indicate the transition to a new character or word. This step ensured that comparisons could be made on a per-character basis—e.g., the character “萬” from one sample of the Chinese phrase would be explicitly compared to “萬” in another sample. Each digit in the numeric sequence was similarly treated as a separate unit for analysis.

#### 2.2.2. Feature Extraction

After segmentation, each sub-signature (characters or digits) with *n* sample points was extracted with twenty features as defined in Table 2, as in [23]. The features were chosen to capture kinematic and dynamic characteristics that are informative for intra- and inter-writer discrimination.

#### 2.2.3. Z-Score Standardization

Due to the variability in handwriting scale and writing shift, the values of the extracted feature were standardized to Z-scores. Each feature value $f_{k}(i)$ was standardized by the following equations:

(1) $$f_{k}^{z}(i)=\frac{f_{k}(i)-mean(f_{k})}{\sqrt{\sum_{j=1}^{n}{\left[f_{k}(j)-mean(f_{k})\right]}^{2}/n}},$$

(2) $$mean(f_{k})=\left(\sum_{j=1}^{n}f_{k}(j)\right)/n,$$

where $f_{k}(i)$ denotes the *k*-th feature value of the *i*-th sample point.

**Figure 2:**
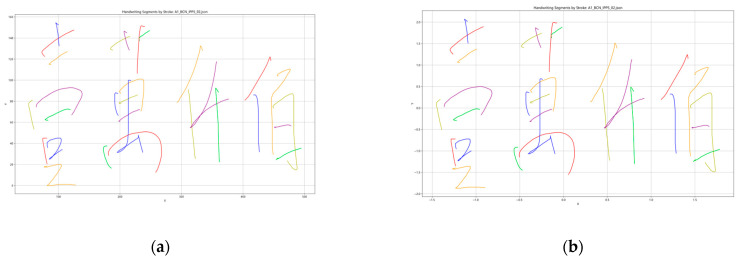
An example that represents the X-coordinate and Y-coordinate feature values of Chinese handwriting before and after z-score standardization: (**a**) Before z-score standardization; (**b**) After z-score standardization.

An example of Chinese handwriting before and after z-score standardization is shown in Figure 2. The result of preprocessing was that each signature stroke of data was centered and ready for time-series comparison.

### 2.3. Matching

Recently, DTW-based methods have been widely applied as a matching technique in on-line signature verification. The DTW method aligns two handwriting signatures by locally compressing or expanding the time axis of the two temporal functions. In this study, the FastDTW method and Shape Context Dynamic Time Warping (SC-DTW) are employed as matching techniques for handwriting verification.

#### 2.3.1. Dynamic Time Warping (DTW)

We first introduce the original DTW to compare pairs of signature time series. Given two signatures, *S*1 and *S*2, with lengths *n* and *m*, respectively, DTW seeks an optimal alignment path through their time-frequency matrix that minimizes the cumulative distance between the sequences. To calculate the shortest alignment path between two sequences, a dynamic programming (DP) algorithm is used to find the optimal alignment path by minimizing the cost function between points in the permutation sequence. The dynamic programming algorithm is briefly described as follows:

1. Create a cost matrix: $C\in R^{n\times m}:C_{l}=\sum_{o=1}^{20}{\left(f_{o}^{z}(S1(i))-f_{o}^{z}(S2(j))\right)}^{2},i\in \left[1,n\right],j\in \left[1,m\right]$ represents the location cost of the pairwise distances between *S*1(*i*) and *S*2(*j*).
2. Find the aligned path that traverses the low-cost region (shortest distance): The aligned path is a sequence of points (AP(1), AP(2), …, AP(*k*)) constructed by dynamic time correction, where AP(l) = (*S*1(*i*), S2(*j*)) ∈ [1, *n*] × [1, *m*]. For l ∈ [1, *k*], and satisfy the following conditions:
  A. Boundary condition: AP(1) = (*S*1(1), *S*2(1)) and AP(*k*) = (*S*1(*n*), *S*2(*m*)).
  B. Monotonically increasing condition: The time sequences of two aligned sequences must be monotonically increasing.
  C. Step size condition: This criterion limits the length of jumps (temporal offsets) in the aligned sequence of the path.
3. Optimal path: The path with the minimum cost of alignment is referred to as the optimal alignment path. That is, the cost function of the alignment path calculated from all pairwise distances is $C_{P}=\sum C_{l}$ and finding the path with the minimum cost $C_{min}$ is the optimal alignment path. Then, the average cost *D*, which represents the average distance between the two time-correlated sequences, is defined as follows.
  (3) $$D=\frac{C_{min}}{\sqrt{n\times m}}.$$

**Figure 3:**
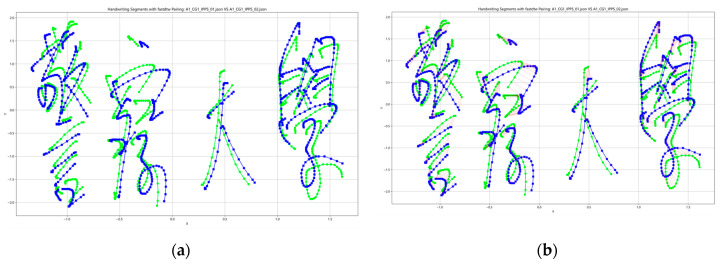
An example of aligning a pair of Chinese characters before and after alignment by FastDTW, the green lines mean the first time handwriting sample, and the blue lines mean the second one: (**a**) Before alignment; (**b**) The red lines mean the aligned pairs after alignment.

As described earlier, the classic DTW algorithm has quadratic time complexity. In contrast, FastDTW is a linear-time and space-complexity method that recursively projects a solution from a coarse resolution and refines the projected solution. We implement the FastDTW algorithm [20] for handwriting verification in this study. An example of aligning a pair of Chinese characters using FastDTW is shown in Figure 3.

#### 2.3.2. Shape Context Dynamic Time Warping (SC-DTW)

In parallel, we performed an analysis using Shape Context Dynamic Time Warping (SC-DTW), an advanced variant of DTW. SC-DTW modifies the distance measure between points by comparing shape context descriptors rather than raw coordinate-pressure values. To implement this, we computed a shape context histogram for each feature of each sample point in a signature. This was achieved by considering the point as the origin and examining the relative positions of all other points in the same signature. The histogram captures the distribution of points around the reference point in a binned radial and angular space. In practice, we used a moderately sized shape context (10 radial bins for 20 features, as shown in Table 2) to describe the local shape around each point.

When comparing two signatures using SC-DTW, we define the distance between two points *S*1(*i*) and *S*2(*j*) as the ${SCC}_{l}$ distance between their shape context histograms. The ${SCC}_{l}$ distance (as the $C_{l}$ in FastDTW) is defined as follows:

(4) $${SCC}_{l}=\frac{1}{2}\sum_{o=1}^{20}\sum_{k=1}^{10}\frac{{\left[h_{k}(f_{o}^{z}(S1(i)))-h_{k}(f_{o}^{z}(S2(j)))\right]}^{2}}{h_{k}(f_{o}^{z}(S1(i)))+h_{k}(f_{o}^{z}(S2(j)))}.$$

The use of the ${SCC}_{l}$ metric is common for comparing distributions (it ranges from 0 when histograms are identical, and increases as they differ). With this new pointwise distance, the DTW algorithm was applied in the same manner as before to find an optimal alignment path. Thus, SC-DTW still produces a cumulative distance, but one that reflects not just point-by-point location differences but broader shape context differences.

SC-DTW was applied to the same set of signature pairings as regular DTW. We hypothesized that SC-DTW might improve inter-writer discrimination, especially because different people often have qualitatively different stroke shapes that the shape context can capture. In contrast, a FastDTW might sometimes be fooled if two different writers produce similarly looking trajectories with varying parameterizations of time. It could also potentially lower intra-writer distances by finding better alignment for shape-wise similar segments.

### 2.4. Evaluation Metrics

To compare the performance of FastDTW and SC-DTW in distinguishing writers, we looked at several metrics:

- Distance Distributions: We summarized the distribution of distances for intra-writer vs. inter-writer comparisons under each method. The separation between these distributions is a statistical indicator of how well the technique can distinguish between same-writer and different-writer cases.
- Summary Statistics: For each distribution, we calculated the mean and standard deviation of distances. In addition, Levene’s test, Welch’s t-test, and Student’s t-test are applied to test the variances and the means between distributions.
- Overlap and Error Rates: We computed the degree of overlap between the intra- and inter-writer distance distributions. If we treat this as a binary classification problem (same-writer vs. different-writer), an ideal scenario is zero overlap (a threshold can cleanly separate the two).
- Device Comparison: We also stratified the results by device. For each device, we examined the intra-writer distance distribution for signatures collected on that device, as well as for inter-writer comparisons (where each pair involved signatures from the same device type). We then compared these across devices to see if one device yielded consistently lower or higher distances (indicating more or less stable capture).

It is worth noting that all analyses were conducted using custom Python 3.11 scripts and libraries (e.g., z-score standardization and our own implementation of SC-DTW, based on open-source shape context code).

## 3. Experimental Results

This section describes the detailed experimental process, including the creation of the dataset and the presentation of experimental results.

### 3.1. Dataset

This study collected handwriting samples from 30 adult participants under five different types of writing conditions. The five categories are writing with a stylus on a Samsung Galaxy Tab (SPPS), writing with an Apple Pencil Pro on an Apple iPad Pro (IPPS), writing with an Apple Pencil on an Apple iPad mini (IPMS), writing with a finger on a Samsung Galaxy Tab (SPPF), and writing with a finger on an Apple iPad Pro (IPPF). Due to the required handwriting diversity, each person provided writing samples from 11 different types (including Chinese characters, English characters, and numbers shown in Table 3). For every type of writing sample, 10 samples were collected.

Additionally, two custom handwriting capture applications were developed for each platform (iOS and Android) to record signatures. The writing area corresponded to a physical size of approximately 100 × 50 mm on screen. During the handwriting capture experiment, participants should go to a comfortable location on their own and obtain the information according to the written template of this study. After data collection, a total of 16,500 DCS samples were received and stored in the ISO/IEC 19794-7:2021 [2] data format.

### 3.2. Experimental Results and Analysis

#### 3.2.1. Comparison Between the FastDTW and SC-DTW Methods

Performance evaluations of FastDTW and SC-DTW methods are first conducted. The distribution of distances for various comparisons under each method is shown in Table 4. According to Table 4, FastDTW has a smaller average distance in all group types. The separation between these distributions is a statistical indicator of how well the FastDTW can distinguish between intra-writer and inter-writer cases. In addition, the test results (Table 5) of Levene’s test, Welch’s *t*-test, and Student’s *t*-test between distributions also show that both methods are significantly efficient in distinguishing intra-writer and inter-writer groups. It also demonstrates that FastDTW is more effective in distinguishing between handwriting samples from intra-writer and inter-writer contexts.

Additionally, we compare the time complexity of FastDTW and SC-DTW, presenting the evaluation results in Table 6. The test platform’s specifications include an AMD Ryzen 7 7735HS processor with Radeon Graphics (3.20 GHz), an NVIDIA GeForce RTX 4060 Laptop GPU, and 32 GB of RAM (4800 MT/s), assembled in China. From Table 6, we can see that the speed of FastDTW is approximately 2.5 times faster than that of SC-DTW.

#### 3.2.2. Comparison of Writing Conditions

**Figure 4:**
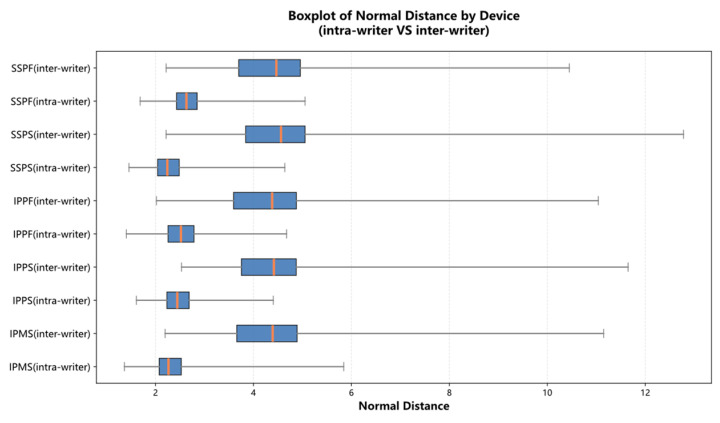
The stability of devices between intra-writer and inter-writer.

In this subsection, we focus on the impact of the writing device and conditions. The average distance can determine the stability of devices between intra-writer and inter-writer. The results are shown in Table 7. Figure 4 is a visual representation of data aligned by the FastDTW method in Table 7. From Table 7 and Figure 4, we can see that all means and standard deviations of the average distance under the intra-writer condition are lower than those under the inter-writer condition. In addition, in the intra-writer group, the means and standard deviations on the finger writing condition (IPPF and SPPF) are larger than those on the stylus writing condition. To determine the difference in stability under stylus and finger writing conditions, Levene’s test and the Z-test are used to obtain the statistical significance (Table 8).

Then, the differences between writing on the same device and writing across devices were evaluated. Table 9 shows the average distance between writing on the same device and writing across devices. According to Table 9, the average distances that are written across devices are larger than those written on the same device. To determine the difference between writing on the same device and across devices, Levene’s test and the Z-test are used to obtain the statistical significance (Table 10).

## 4. Discussion

This study provides a systematic evaluation of digitally captured handwriting under different devices, writing tools, and temporal alignment algorithms. Several key observations emerged from our experiments, offering insights into both the behavioral characteristics of digital handwriting and the methodological considerations necessary for its forensic application.

### 4.1. Influence of Writing Tools and Devices on Handwriting Stability

One of the clearest findings is the significant difference in handwriting stability between stylus-based writing and finger-based writing across all devices. Stylus input produced consistently lower intra-writer distances and more precise separation between intra-writer and inter-writer distributions, regardless of the alignment method used. A plausible explanation is that styluses provide a stable point of contact, higher friction consistency, and access to pressure and tilt channels, which allow for richer and more repeatable dynamic features. In contrast, finger input lacks fine-point precision and can introduce irregular contact areas, occlusion effects, and reduced control due to screen surface smoothness, leading to greater variability.

Moreover, device-specific factors such as sampling rate, stylus sensitivity, and hardware latency likely contributed to the observed differences in stability. For instance, the Apple Pencil Pro and S-Pen utilize dedicated hardware protocols that provide fine-grained pressure and tilt measurements. In contrast, finger input relies on capacitive touch sampling, which typically has lower temporal resolution. These hardware-level differences underscore the importance of maintaining consistent acquisition conditions, as supported by recommendations found in the ENFSI Best Practice Manual and ANSI/ASB guidelines.

### 4.2. Same-Device vs. Cross-Device Comparison Challenges

Our results further demonstrate that writing samples collected on different devices exhibit substantially higher alignment distances than those obtained on the same device. This discrepancy can be attributed to multiple factors, including device-specific sampling rates, variations in coordinate system scaling, and differences in sensor hardware or stylus characteristics. Although preprocessing with z-score normalization reduces global variability, device-induced differences in stroke dynamics and pressure sampling appear to persist.

Table 9 and Table 10 demonstrate that the “across devices” distances are higher than the “same device” distances, and statistical tests indicate these differences are significant. These findings suggest that cross-device DCS comparisons should be approached with caution in forensic practice. While dynamic features can still provide meaningful discriminatory information, their reliability diminishes when comparing samples captured under heterogeneous acquisition conditions. Importantly, this variability reinforces the forensic guideline that questioned and reference signatures should be collected using comparable tools whenever possible.

### 4.3. Algorithmic Performance and Implications for Forensic Use

Across all scenarios, although SC-DTW was hypothesized to improve inter-writer discrimination by incorporating shape context information, the experimental results did not support this hypothesis in the present dataset. Instead, FastDTW demonstrated better separation SC-DTW in terms of distance separation and computational efficiency under the examined conditions. The superiority of FastDTW may stem from its robustness in handling time-series variations typical of handwriting, whereas SC-DTW’s reliance on shape context descriptors makes it more sensitive to noise, segmentation errors, or distortions introduced by hardware inconsistencies. FastDTW aligns multichannel function features (including position/pressure/velocity-like channels after standardization), which can remain informative even when local geometric distributions shift across devices. This may help explain the more apparent separation observed between intra- and inter-writer groups in our dataset. In cross-device DCS data, differences in sampling density, coordinate quantization, and capture latency can distort local spatial distributions, making shape-context descriptors more sensitive to hardware-induced variations than direct feature-wise alignment. This can reduce its practical advantage in mixed-device settings. While SC-DTW has previously demonstrated competitive performance in machine-learning-based biometric verification systems, its benefits may not translate directly to mixed-device forensic datasets, where noise patterns differ significantly from those in controlled public datasets.

Table 6 shows the runtime evaluation table between the two methods. FastDTW has a lower average time per alignment, and it was explained that SC-DTW introduces additional overhead due to the computation and comparison of histogram descriptors at each time point.

The comparison between FastDTW and SC-DTW is based not on the absolute magnitude of distance values across methods, but rather on their relative discriminatory ability within each method, specifically the separation between intra-writer and inter-writer distance distributions, as well as computational efficiency. Each method employs a distance function appropriate to its feature representation, and comparisons are made within the method rather than through cross-metric normalization.

From a forensic standpoint, the interpretability of DTW-based methods is an advantage. Examiners can visualize alignment paths and understand distance components, making FastDTW a practical choice for supporting human expert analysis. However, the algorithm does not directly incorporate writer-specific behavioral models, suggesting that combining FastDTW with statistical or machine-learning classifiers may improve robustness, particularly in cross-device contexts.

### 4.4. Limitations of the Present Study

Despite its strengths, several limitations should be acknowledged. First, the participant pool consisted of 30 individuals, which, although sufficient for initial analysis, limits the generalizability of the findings to broader populations. Second, the study focused solely on natural handwriting variability and did not incorporate simulated or skilled forgeries, which is an essential consideration for forensic casework. Third, only three tablet models and their corresponding styluses were included. Additional commercial signature pads, mobile phones, and high-frequency capture devices may exhibit different behaviors. Lastly, the analysis focused on character-level segmentation, which may not fully reflect real-world signing behavior where entire signatures or words are treated as holistic entities.

### 4.5. Future Research Directions

Future work should expand the dataset to include larger and more diverse groups, as well as samples collected from a broader array of devices and pen technologies. Incorporating participants from diverse ages, educational, cultural, and regional backgrounds improves cross-cultural generalizability, reduces potential confounding, and applicability in international forensic contexts. In addition, incorporating controlled forgery experiments—both simple imitation and skilled forgeries—would provide deeper insights into the discriminative power of DCS features under adversarial conditions. Moreover, exploring feature selection techniques and integrating deep learning models with DTW-based alignment could improve both accuracy and robustness. Device-specific normalization strategies or domain adaptation techniques may also help reduce cross-device discrepancies. Ultimately, developing a standardized cross-device calibration framework may be critical for enabling reliable DCS comparisons in forensic and biometric applications.

## 5. Conclusions

A large-scale dataset of 16,500 digitally captured handwriting samples, obtained using three commercial tablets under five different writing conditions, is constructed to investigate the stability of Digital Capture Signature (DCS) data. Additionally, we evaluate the performance of two notable time-series matching algorithms. The experimental results demonstrate that FastDTW consistently achieves lower intra-writer distances, better separation between intra-writer and inter-writer distributions, and approximately 2.5 times faster computation than SC-DTW. These findings suggest that FastDTW is a more effective and efficient technique for DCS-based handwriting comparison within the examined contexts under the examined conditions. The analysis further reveals that handwriting produced with a stylus exhibits significantly higher stability and discriminative power than handwriting produced with a finger, across all devices. Additionally, we demonstrate the difference between writing on the same device and across multiple devices. This observation supports existing recommendations in the ENFSI and ANSI/ASB standards, which emphasize collecting reference signatures using the same device type and writing conditions as the questioned signatures. The present results also highlight that device- and tool-dependent factors can considerably influence the dynamic characteristics of digital handwriting, underscoring the importance of standardized acquisition conditions in forensic and biometric applications.

This study provides empirical evidence and methodological insights that may inform the development of future guidelines for digital signature examination and the design of secure electronic signature systems in financial, insurance, and governmental sectors. In particular, our dataset and findings offer a foundation for improving cross-device comparability and enhancing the forensic value of dynamic signature features.

Several limitations should be noted. The sample size, although adequate for observing clear trends, remains limited to 30 participants. The study also focuses on three tablets and their native styluses, without including a broader range of commercial signature pads or high-frequency capture devices. Moreover, the experiments examined natural handwriting stability but did not incorporate forgery scenarios. These factors may constrain the generalizability of the results.

Our future work may extend the dataset to include larger and more diverse populations, additional device types, and controlled attempts at forgery. Further research integrating machine learning models with DTW-based features may also aid in the development of automated assessment tools for digital signature verification. We anticipate that the present dataset, workflow, and results will contribute to the continuing advancement of DCS research and support its increasing relevance in forensic science and digital identity verification.

## Figures and Tables

**Table 1:** Summary of DCS data channels and their definitions (as recorded by the capture software).

Channels	Unit	Definition
Time	Second	Time since the first data
X coordinate	Pixel	Horizontal position
Y coordinate	Pixel	Vertical position
Pressure	None	Pen tip pressure
Altitude Angle	radian	The angle between the pen and the surface, 0~π/2
Azimuth Angle	radian	The angle from the top direction on the surface to the vertical projection of the pen on the surface, 0~2π
Stroke order	None	The stroke order from the pen tip to the screen and back until it leaves.

**Table 2:** Summary of extracted function features.

Category	Description	Definition
Position	X-coordinate	x(i)
	Y-coordinate	y(i)
	Location	$L(i)=\sqrt{x(i{)}^{2}+y(i{)}^{2}}$
	Change in X-coordinate	$\Delta x_{i}=x(i+1)-x(i)$
	Change in Y-coordinate	$\Delta y_{i}=y(i+1)-y(i)$
	Change in location	$\Delta L(i)=\sqrt{(\Delta x(i){)}^{2}+(\Delta y(i){)}^{2}}$
Pressure	Pressure	p(i)
	Change in pressure	$\Delta p_{i}=p(i+1)-p(i)$
Velocity	X-velocity	$v_{x}[i]=\frac{x(i+1)-x(i)}{t(i+1)-t(i)}$
	Y-velocity	$v_{y}[i]=\frac{y(i+1)-y(i)}{t(i+1)-t(i)}$
	Location velocity	$v(i)=\sqrt{v_{x}^{2}(i)+v_{y}^{2}(i)}$
Acceleration	X-acceleration	$a_{x}[i]=\frac{v_{x}(i+1)-v_{x}(i)}{t(i+1)-t(i)}$
	Y-acceleration	$a_{y}[i]=\frac{v_{y}(i+1)-v_{y}(i)}{t(i+1)-t(i)}$
	Location acceleration	$a(i)=\sqrt{a_{x}^{2}(i)+a_{y}^{2}(i)}$
	Centripetal acceleration	$a_{c}(i)=\left[v_{x}(i)a_{y}(i)-v_{y}(i)a_{x}(i)\right]/v(i)$
Angle	Cosine of the angle between the x-axis and the signature curve	$\cos\alpha (i)=\frac{x(i+1)-x(i)}{\sqrt{{\left[x(i+1)-x(i)\right]}^{2}+{\left[y(i+1)-y(i)\right]}^{2}}}$
	Sine of the angle between the x-axis and the signature curve	$\sin\alpha (i)=\frac{y(i+1)-y(i)}{\sqrt{{\left[x(i+1)-x(i)\right]}^{2}+{\left[y(i+1)-y(i)\right]}^{2}}}$
	Cosine of the angle between the x-velocity and the location velocity	$\cos\beta (i)=v_{x}(i)/v(i)$
	Angle between the x-axis and the signature curve	$\theta (i)=\tan^{-1}\frac{y(i+1)-y(i)}{x(i+1)-x(i)}$
	Angle velocity	$v_{\theta }(i)=\tan^{-1}\frac{\theta (i+1)-\theta (i)}{t(i+1)-t(i)}$

**Table 3:** Eleven writing types.

Chinese Characters	English Characters	Number Digits
Name 1	Name 1	Phone number
Name 2	Name 2	ID template
Name 3	Name 3	
Chinese number		
Organization Name 1		
Organization Name 2		

**Table 4:** Various comparisons of the distribution of distances for each method.

Group Types	FastDTW		SC-DTW	
	Mean	Std. *	Mean	Std. *
intra-writer	2.3541	0.1918	4.9372	0.6352
inter-writer	4.3055	0.5573	7.2826	0.7226
inter-device	3.1410	0.2487	6.2834	0.6449

* Std. denotes the standard deviation of the average distance.

**Table 5:** The results of Levene’s and Welch’s *t*-test for each method.

Group Types	Levene’s Test ^a^		Welch’s *t*-Test ^b^	Student’s *t*-Test ^c^
	FastDTW	SC-DTW	FastDTW	SC-DTW
intra-writer vs. inter-writer	0.0486	0.2185	2.4323 × 10^−11^	9.1275 × 10^−4^

^a^ Levene’s test is a statistical procedure used to assess the homogeneity of variances (also known as homoscedasticity) across two or more groups. ^b^ Welch’s *t*-test (also called Welch’s unequal variances *t*-test) is a statistical method used to compare the means of two independent groups when the assumption of equal variances is violated. ^c^ Student’s *t*-test is a statistical method used to compare the means of two independent groups under the assumption of equal variances.

**Table 6:** The time cost evaluation for each method.

	FastDTW	SC-DTW
Average time per alignment (s)	0.000769697	0.001923232

**Table 7:** The average distance between intra-writer and inter-writer under different devices.

Writing Conditions		FastDTW		SC-DTW	
		Mean	Std. *	Mean	Std. *
intra-writer	IPMS	2.33	0.21	2.33	0.21
	IPPF	2.53	0.22	2.81	0.25
	IPPS	2.48	0.19	2.48	0.19
	SPPF	2.66	0.20	2.95	0.22
	SPPS	2.29	0.19	2.29	0.19
inter-writer	IPMS	4.46	0.59	7.52	0.72
	IPPF	4.39	0.57	7.56	0.82
	IPPS	4.48	0.54	7.61	0.70
	SPPF	4.48	0.57	7.44	0.76
	SPPS	4.59	0.63	7.78	0.77

* Std. denotes the standard deviation of the average distance.

**Table 8:** The test results for stability under stylus and finger writing conditions.

Writing Conditions	Levene’s Test	Z-Test
stylus vs. finger	0.1660	0

**Table 9:** The average distance between writing on the same device and writing across devices.

Writing Conditions		FastDTW		SC-DTW	
		Mean	Std. *	Mean	Std. *
the same device	stylus	2.37	0.19	5.19	0.64
	finger	2.59	0.21	6.42	0.72
across devices	stylus	3.15	0.31	5.06	0.70
	finger	3.47	0.18	6.76	0.58

* Std. denotes the standard deviation of the average distance.

**Table 10:** The test results for writing on the same device and across devices.

Writing Conditions	Levene’s Test	Z-Test
stylus	0.1356	0
finger	0.1472	0

## Data Availability

The raw data supporting the conclusions of this article will be made available by the authors on request.

## Abbreviations

The following abbreviations are used in this manuscript:

DCS	Digitally captured signature
FastDTW	Fast dynamic time warping
SC-DTW	Shape context dynamic time warping
